# Metallic Nitride Microfluidic e‑Tongue: A Novel Selective Approach for the Detection of Macronutrients in Soil

**DOI:** 10.1021/acssensors.5c00921

**Published:** 2025-08-12

**Authors:** Carla D. Boeira, Leonardo M. Leidens, Endel E. Carvalho Costa, Maria H. Gonçalves, Amanda S. Perillo, Felipe A. Ferraz, Maria C. Marchi, Flavio M. Shimizu, Lucas R. Amaral, Fernando Alvarez, Antonio Riul

**Affiliations:** † Instituto de Física ‘Gleb Wataghin’ (IFGW), 28132 Universidade Estadual de Campinas (UNICAMP), Campinas, São Paulo 13083-970, Brazil; ‡ Universidad de Buenos Aires, Facultad de Ciencias Exactas y Naturales, Departamento de Química Inorgánica, Analítica y Química Física, Buenos Aires C1428EGA, Argentina; § CONICET - Universidad de Buenos Aires, Instituto de Física de Buenos Aires (IFIBA), Centro de Microscopías Avanzadas (CMA), Buenos Aires C1428EHA, Argentina; ⊥ School of Agronomic Engineering (FEAGRI), Universidade Estadual de Campinas (UNICAMP), Campinas, São Paulo 13083-875, Brazil

**Keywords:** precision agriculture, e-tongue, GLAD, nitrides, supervised learning methods

## Abstract

The growing global demand for food requires optimizing agricultural practices and more rational use of natural resources without expanding cropping areas. Precision agriculture (PA) tools are essential for accurately applying fertilizers and herbicides, reducing costs, and avoiding environmental impacts. Standard macronutrient mapping methods are costly and time-consuming, limiting denser sampling collection in the field. Consequently, devices employing new materials with specific properties matching sensitivity to agricultural nutrients, robustness to face intensive climatic variations, and economical manufacturing viability are mandatory. In this sense, microfluidic impedimetric e-tongues have emerged as practical tools in PA due to their high sensitivity, adaptability, affordability, and ease of use. These sensors provide rapid qualitative and quantitative results in liquid media, with applications extending to food analysis, environmental monitoring, and biosensing. Here, metallic nitride thin films (CrN, BN, and TiN) deposited via physical vapor deposition (PVD) using the glancing angle deposition (GLAD) technique are applied as sensing units presenting high sensitivity, controlled micro- and nanostructures, durability, reproducibility, and mechanical robustness, essential characteristics for future on-site soil analyses. The GLAD technique allows precise control over the micro- and nanostructures deposited on gold interdigitated electrodes to create molecular sieves for a possible capture of target species (e.g., K^+^, Na^+^, Mg^2+^, Ca^2+^, PO_4_^3–^) from soil samples. We demonstrate the feasibility of using distinct nitrides as sensing units in a microfluidic e-tongue tested with soil samples with distinct compositions diluted in water without pretreatments. The sensor successfully differentiated all samples tested, showing higher K and Mg macronutrient resolution. These findings demonstrate the high potential to detect minute changes (<1 mmol·L^–1^) in soil fertilization, with results compared by four prediction models, paving the way for future in situ analyses envisaging a controlled delivery of macronutrients during fertilization.

## Graphical abstract

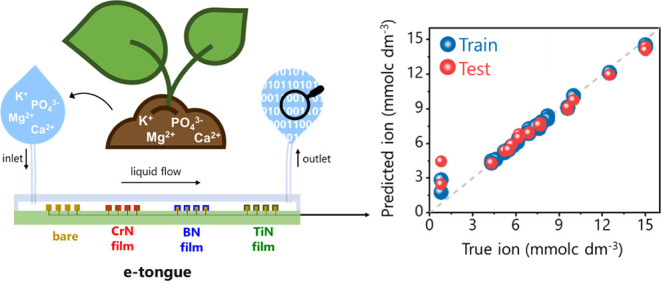

## Introduction

Population growth drives global demand for food, while the productivity of cropped areas remains below their worldwide yield potential.^1^ Although agricultural fields are expected to grow in the coming decades as a strategy to increase food production, the associated environmental impacts further aggravate this situation.^2^ Modern agricultural practices are increasingly focused on enhancing resource management through improved efficiency, productivity, quality, profitability, and sustainability, all while addressing the escalating global demand for food and mitigating environmental challenges such as deforestation and land degradation.^3,4^ Since the 90s, precision agriculture (PA) has emerged as a transformative approach, driving substantial productivity advancements by optimizing the utilization of agricultural inputs. Nevertheless, conventional soil data collection methods remain impractical, constrained by prohibitively expensive, labor-intensive sampling processes and protracted laboratory analyses.^3,5^ The need to monitor nutrient supply information in the soil has led to innovative technologies designed to rapidly, affordably, and autonomously capture high-resolution spatiotemporal soil data.^5,6^ In response, PA has catalyzed the emergence of advanced strategies that enhance crop management, optimize nutrient delivery, and enable precision soil fertilization through intelligent sensing systems underpinned by interdisciplinary synergies across diverse scientific disciplines.^7,8^

Microfluidic impedimetric e-tongues are promising analytical tools that fit some of the above requirements due to their high sensitivity, adaptability, and successful usage in food analysis, environmental monitoring, and medical diagnostics.^9–11^ A standard microfluidic impedimetric device incorporates microchannels through which a liquid sample flows over active sensor surfaces (sensing units).^12^ The sensor arrangement detects minute impedance variations resulting from molecular interactions at the electrode–electrolyte interface, while advanced multivariate data analysis algorithms enable the real-time discrimination, classification, and quantification of target analytes.^12,13^

Significant advances in surface engineering focus on material properties for broader applications, looking for improvements in sensitivity, reproducibility, hardness, and electrical and mechanical robustness properties in sensors. The glancing angle deposition (GLAD) technique uses oblique angle deposition, enabling nanostructured metal nitrides^14,15^ with tailored film thickness, bulk geometry, and surface roughness^16–18^ to act as the sensing elements in an impedimetric e-tongue devoted to soil analyses. Here, chromium nitride (CrN), boron nitride (BN), and titanium nitride (TiN) nanostructured films are engineered and deposited onto gold interdigitated electrodes (IDE) as active sensing layers via the GLAD technique. These materials are evaluated for their analytical performance in detecting critical soil nutrients—specifically K^+^,Na^+^,Mg^2+^,Ca^2+^, and PO_4_^3–^—within aqueous soil suspensions. In order to establish a reference library to calibrate the electronic tongue (e-tongue) system through comprehensive physicochemical characterization, a data set consisting of 26 soil samples systematically collected from various locations within a georeferenced agricultural area was used. The detailed characterization of these samples, including impedance data and physicochemical parameters, is essential for the development of predictive models. The objective is to predict the concentrations of macronutrients such as potassium (K^+^), magnesium (Mg^2+^), calcium (Ca^2+^), and phosphate (PO_4_^3–^) through a machine learning (ML) approach that integrates these data into a multivariate regression model. The study evaluates the effectiveness of different supervised learning methods, including partial least squares, decision tree, random forest, and XGBoost, with a focus on external validation using blind samples of unknown composition. The random forest model achieved the best results, demonstrating high accuracy in predicting elemental concentrations, particularly for potassium and magnesium, suggesting a specific correlation with nanocage structures formed in nitride compounds. These findings may open new possibilities for the use of electronic sensing technologies in soil analysis, contributing to the advancement of precision agriculture.

## Experimental Section

### Nitride Thin Film Deposition

The controlled deposition of the CrN, BN, and TiN films on electroless nickel immersion gold (ENIG) interdigitated electrodes (IDEs) linearly arranged in a printed circuit board (PCB) is crucial in forming the microfluidic impedimetric e-tongue. The same films were deposited on silicon substrates in the same batch for thin-film characterization. The IDEs are plated on PCBs acquired from TEC–CI *Circuitos Impressos* (São Paulo, SP, Brazil), with four pairs of digits having 5 mm length, 0.2 mm width, and 0.2 mm spacing from each other. Three IDEs are coated with nitride films, while one IDE remains nonmodified for comparison purposes (Figure 1). The film deposition uses multipurpose equipment with an RF sputtering source, Kurt J. Lesker Company, Model RF300, operating at 13.6 MHz with a maximum power of 300 W and a target 2″ diameter. This system also includes a modified sample holder allowing angle variation during deposition (GLAD), with the PCB placed in the sample holder ∼6 cm away from the target. The chamber is evacuated to a back pressure of ∼1.7 × 10^–4^ Pa before deposition by a combination of mechanical and turbomolecular pumps (Edwards, Next 400 Model). During film deposition, a constant flow mixture atmosphere of 2:3 argon and nitrogen gases is controlled by mass flowmeter controllers (MKS, Series 600). The working pressure of ∼1 Pa is kept constant through a downstream throttle automatic control system. The nominal RF power for deposition is set at 150 W for the titanium (Ti) and chromium (Cr) targets and 75 W for the boron nitride (BN) targets. All films are deposited at room temperature (30 °C), and at the beginning of the process, the sample underwent an oscillatory movement, shifting from directly in front of the target to an angle of 85° in front of the radio frequency source. The automatic control of the substrate support movement allows atoms to land on the substrate at different angles to achieve the desired nanostructure formation. Deposition times are previously selected based on the sputtering rate to obtain films with similar thicknesses. Figure 1 illustrates the nitrides deposited on the IDEs on the PCB and a schematic of a 500 μm width poly­(dimethylsiloxane) (PDMS) microchannel placed on the sensing units.

**Figure 1:**
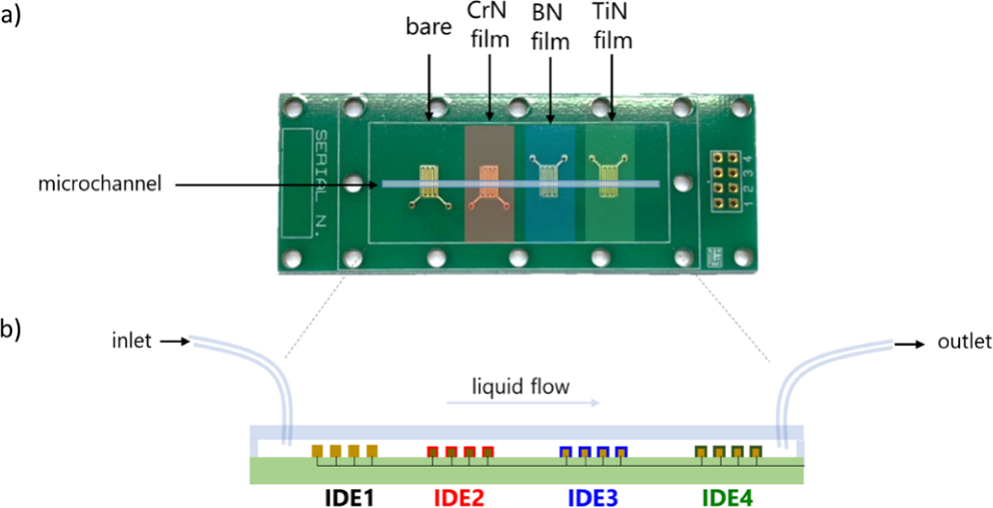
(a) Printed circuit board (PCB) representation with a bare IDE (IDE1) and three IDEs coated with CrN (IDE2), BN (IDE3), and TiN (IDE4) nitride films; (b) cross-section representation of the device.

### Thin Films Characterization

The cross-sectional structure and thickness of the films are obtained by using a field emission scanning electron microscope (FE-SEM, Zeiss Supra 40) to verify the deposited film structures. The chemical bonding and composition of thin films are determined *ex situ* by X-ray photoelectron spectroscopy (XPS) using the Thermo Alpha 110 Hemispherical Analyzer, with 1486.6 eV photons from an Al target (Kα line). The XPS pressure in the analysis chamber is <8 × 10^–7^ Pa during measurement. The system is calibrated using a clean sputtered Ag standard sample *in situ*, referencing the binding energy of electrons in 3d^5/2^ orbitals at 368.2 ± 0.1 eV. The Shirley method is applied to remove the electron inelastic collision spectral background. The quantification of element concentrations is performed using Thermo Advantage software, with deconvolution carried out using Casa Software.^19^

### Microfluidic e-Tongue Impedance Measurements and Analysis

Polydimethylsiloxane (PDMS) is used in a 3D-printed mold to form a microchannel having ∼500 μm in width and height, and 4 cm length. After curing, the PDMS microchannel crosses four colinear IDEs, as shown in Figure 1, with inlet and outlet made with a biopsy punch.^20^ The electrical impedance measurements are conducted at 25 mV AC voltage in the 1 to 10^6^ Hz frequency range using a Solartron 1260A phase gain/impedance analyzer. This design facilitates rapid multiplexing between all sensing units, with minimal operator interventions (see Figure S1).^21^ Impedance spectroscopy is a powerful tool for analyzing potential interactions at the electrode/electrolyte interface. The procedure is repeated in five independent measurements for each analyte tested to guarantee the statistical reliability. The operator’s role is limited to configuring the electronic setup, changing the analyte, and cleaning the microchannel with 5 mL of ultrapure water (18.2 MΩ·cm) between each measured sample to prevent cross-contamination. The computer-controlled system conducts impedance measurements with samples injected at 15 mL h^–1^ using a syringe pump (New Era Pump System, Model NE-4000). The instrumentation setup is detailed in Figure S1. Tests are conducted in ultrapure water (18.2 MΩ·cm), 1.0 mmol of L^–1^ KCl, and 1.0 mmol of L^–1^ NaCl solutions. Subsequently, soil samples with different macronutrient concentrations, described in supporting information, Table S1, are diluted in ultrapure water at 1 mg mL^–1^ without pretreatment and placed in an ultrasonic bath for 30 min to avoid clogging the microchannel. The soil samples were kept for 40 days in a greenhouse with conventional dairy irrigation to allow for chemical reactions between the added compounds and the soil. Twenty-six (26) soil samples were then collected and submitted to traditional chemical analysis in a commercial laboratory to quantify the macronutrients and further used as a reference to calibrate the microfluidic e-tongue system and to validate the predicted data. In this study, we focused on the sensor response of ions previously quantified through chemical analysis (K^+^, Ca^2+^, Mg^2+^, and P (as PO_4_^3–^)), as described in Table S1. Although other relevant agricultural nutrients, such as NH_4_^+^, NO_3_^–^, and SO_4_^2–^, may also be present, their contributions are not the focus of this discussion due to their dynamics and natural variations in soil matrices. It is important to note here that the presence of possible interferents (NH_4_^+^, NO_3_^–^, and SO_4_^2–^) in the measured samples may interfere with non-Faradaic processes, such as those observed in our systems. However, it is not as critical as in electrochemical processes involving electron transfer, where the competition for adsorption can affect the migration of ions to the electrodes, leading to the formation of complexes, reducing the electrochemical potential due to parallel reactions hindering the reaction kinetics at the electrodes, blocking active sites, or forming complexes, making difficult the detection of the species of interest. Despite the similarities involved, in non-Faradaic processes the presence of interferents can affect the formation of the electric double layer, ionic mobility, and adsorption of species at the electrode/electrolyte interface, as well as the dielectric constant of the medium. Overall, these problems can be circumvented or minimized by using homogeneous, diluted systems, data normalization, identifying optimal frequency regions where the samples are best distinguished (minimizing interface effects >1 kHz), and employing machine learning methods to ensure more reliable results, all of which were utilized in the analyses presented here.

The impedance magnitude is analyzed with unsupervised and supervised methods. At first, clustering algorithms using principal component analysis (PCA) and interactive document mapping (IDMAP).^22,23^ PCA is applied for exploratory observations and pattern recognition, as it compresses data into smaller dimensions without altering the relationships between samples.^24^ Briefly, it is achieved by linearly transforming the original data into a new reference system, known as principal components (PCs). The first PC (PC1) is oriented along the direction of the highest variance of the data, the second one (PC2) is orthogonal to the first, capturing the second largest variance, and so on. The orthogonality of the system guarantees that the information contained in one PC is not present in the other one.^25^ The number of PCs is evaluated based on the amount of variance accounted for and the cumulative percentage of variance. It is also worth mentioning that the original data are initially centered on the mean value and normalized before PCA analysis. The IDMAP technique, available in the PEx-Sensors software toolkit and projection techniques, is applied to visualize the impedance response for each sensing unit in each sample analyzed.^23^ It projects multidimensional data into a 2D space, enhancing its intuitiveness and accessibility. Additionally, the parallel coordinates^23^ technique is included, allowing frequency selection to exclude dimensions that could hinder data discrimination, thus improving the quality of this projection technique. Moreover, this capability is measured through the silhouette coefficient (SC) that ranges from −1 to 1: values below 0 indicate poor classification or lack of significant distinction between samples, while coefficients above 0 suggest the distinction of different samples, indicating better projection quality.^26,27^

We then analyze the data with the supervised machine learning methods briefly outlined below. Partial least squares (PLS) is a statistical method used for modeling relationships between independent (*X*) and dependent variables (*Y*), combining features of PCA and multiple regression. It is generally used in regression and dimensionality reduction, handling highly correlated predictors (multicollinearity) and small sample sizes. It is suitable for high-dimensional data with highly correlated predictors, reducing noise by focusing on latent variables (components) that explain variance in both *X* and *Y*. Decision tree (DT) is a nonparametric supervised learning algorithm for classification or regression that is suitable for simple, interpretable models. It splits data into subsets on feature values, creating a tree-like structure of decisions and coping with numerical and categorical data. The results are easy to understand and visualize; however, they are prone to overfitting, a problem that can be solved by ensemble methods like random forest (RF). An ensemble method is a machine learning technique that combines multiple models to produce a stronger, more accurate, and robust predictive model. In this sense, RF is a learning algorithm that builds multiple decision trees (a forest) and aggregates their results. It trains each tree on a random data set suitable for robust general-purpose modeling. Nodes are split using a random subset of features at each step, with high-accuracy predictions for classification/regression, featuring importance ranking, and handling missing data and outliers. It reduces overfitting compared to single decision trees, being more robust to noise and nonlinear relationships. XGBoost is an optimized gradient-boosting framework designed for speed and performance. It builds trees sequentially, where each new tree corrects errors made by previous ones (boosting) and uses gradient descent to minimize a loss function (e.g., mean squared error). It is recommended for high-dimensional data, being fast and scalable, handling missing values and parallel processing, and maximizing predictive performance in competitions or large data sets. The drawbacks are careful hyperparameter tuning and less interpretable results than those of simpler models. For the above prediction models, we used the same raw impedance data split into training and test data sets in an 80:20 ratio, with an additional 10-fold cross-validation. Macronutrient concentration data from physicochemical analysis served as a reference to calibrate our regression model and validate the predicted blind samples. Out of the 26 soil samples, 23 were used to develop the multioutput regression model, with three samples deliberately excluded to serve as blind tests for assessing the prediction accuracy of macronutrient concentrations based on impedance data.

## Results and Discussions

### Sensor Development

The structural analysis and thickness of the films were determined through cross-sectional SEM images. Figure 2 shows the cross-sectional micrography revealing a columnar structure in the CrN and TiN samples (Figure 2a,c), in contrast to that observed in the BN film in Figure 2b due to magnification. Such behavior is expected, as the GLAD deposition favors columnar structure formation. Moreover, from the cross-sectional images, the film thicknesses are determined to be (52 ± 3) nm, (39 ± 11) nm, and (120 ± 22) nm for CrN, BN, and TiN, respectively.

**Figure 2:**
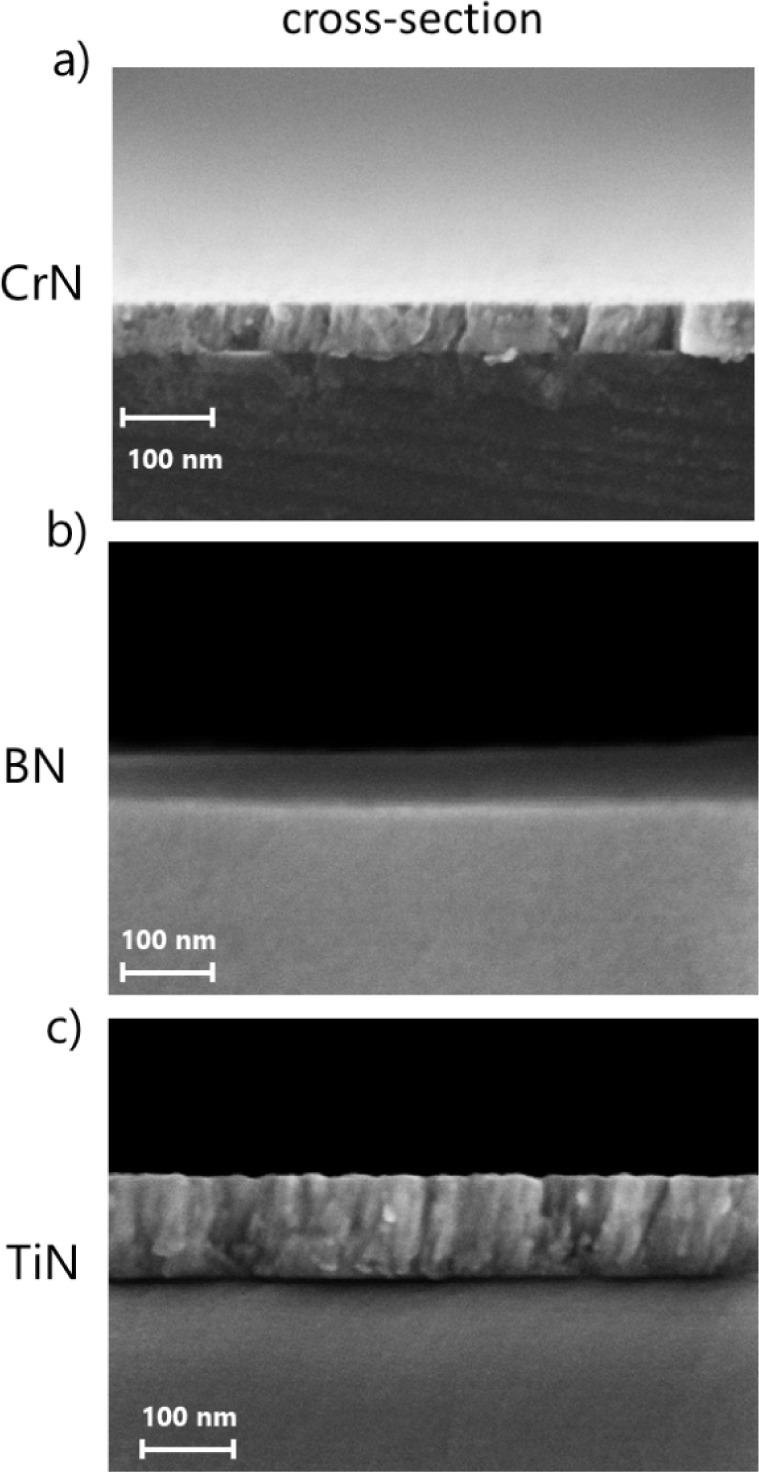
SEM images of the studied samples: cross-sections of (a) CrN, (b) BN, and (c) TiN films.

The structural and chemical bond environment and composition of the films studied are evaluated with high-resolution X-ray photoelectron spectroscopy (XPS), as shown in Figure 3a–f. This technique probes up to ∼10 nm depths from the film surface. Figure 3a,b shows the bands associated with electrons in the Cr 2p and N 1s, respectively. Due to spin–orbit coupling, the Cr 2p band splits into two peaks, i.e., electrons in orbitals Cr 2p^3/2^ and Cr 2p^1/2^ (Figure 3a). The deconvolution of the spectra reveals subpeaks categorized into four other peaks associated with Cr–N and Cr–O bonds. Since our samples are exposed to the atmosphere before XPS analysis, oxygen is expected to be present. Chromium in a nitrogen environment exhibits a ∼575.8 eV binding energy for the Cr–N bond, while the Cr–O bond is located at ∼578.3 eV binding energy.^28,29^ The chemical species of the deconvoluted chromium peaks and the binding energy of the Cr 2p^1/2^ peaks are named in correspondence with the Cr 2p^3/2^ peak. XPS lines, except “s” orbitals, occur in spin–orbit coupling (doublets) named here with Δ*E*.^30^ The Cr–N with Δ*E* = 9.5, and Cr–O with Δ*E* = 9.3 eV.^28^ Figure 3b corresponds to the nitrogen peak deconvolution analysis. The binding energy located at ∼396.8 eV corresponds to the Cr–N bond, while the presence of organic matrices at 399.0 eV might correspond to surface impurities.^29,31^ Figure 3c,d shows the XPS spectra associated with electrons in orbitals B 1s and N 1s. The deconvolution of the band in Figure 3c is displayed in Figure 3d. The components of the deconvolution are attributed to a boron atom bonded to oxygen and nitrogen (B–N_*x*_–O_*y*_) located at 192 eV.^32,33^ In Figure 3d, the peak associated with electrons in the N 1s orbital is deconvolved into two peaks corresponding to B–N at 397.9 and 399.7 eV bonds, while the presence of organic matrices might correspond to surface impurities.^31,34^ Figure 3e,f shows the XPS spectra of the studied TiN samples. Figure 3e shows the band associated with the electrons in orbitals Ti 2p, corresponding to electrons in orbitals Ti 2p^3/2^ (∼455.8 eV) and Ti 2p^1/2^ (∼458.2 eV) due to spin–orbit coupling. Due to the presence of oxygen, Ti–O bonds are evident at an electron binding energy of ∼458.2 eV. The chemical species of the deconvoluted titanium peaks and the binding energy of the Ti 2p^1/2^ peaks are named in correspondence with the Ti 2p^3/2^ peak for Ti–N with Δ*E* = 5.8 eV and Ti–O with Δ*E* = 5.7 eV.^35,36^ The nitrogen peak deconvolution analysis is shown in Figure 3f. The binding energy of 396.9 eV corresponds to the Ti–N bond, while the presence of organic matrices at 399.6 eV corresponds to surface impurities.^37^ Oxygen is also present in residual oxygen gas during growth, depending on the deposition conditions. The XPS spectra for oxygen are presented in Figure S2.

**Figure 3:**
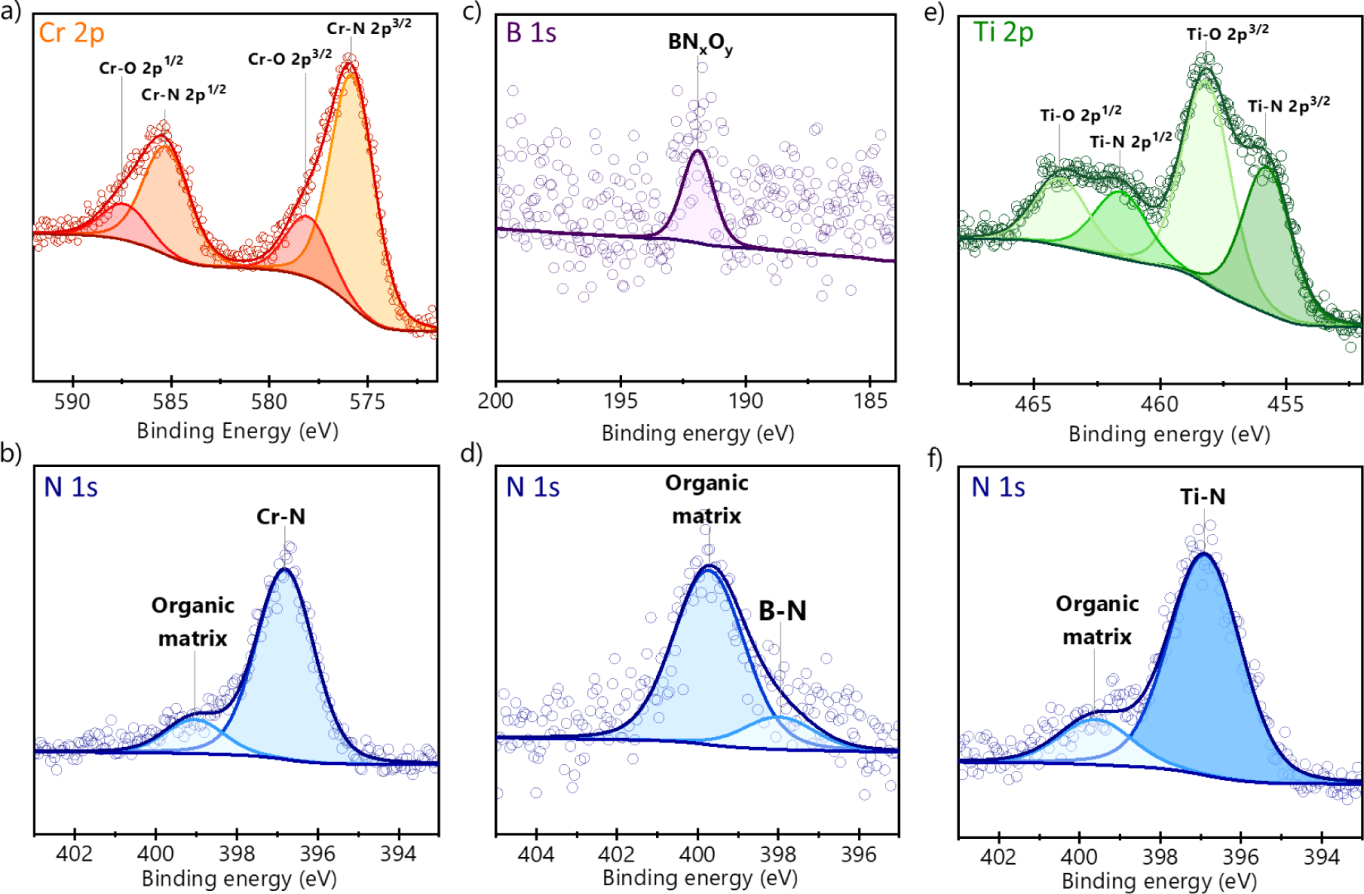
XPS spectra and component deconvolution for the studied films. The composition of the CrN film corresponding to the electrons associated with (a) Cr 2p and (b) N 1s orbitals. The components of the BN bonds electrons associated with (c) B 1s and (d) N 1s orbitals. The components of the electrons are associated with the (e) Ti 2p and (f) N 1s orbitals.

### Sensor Performance

In impedance spectroscopy at lower frequencies (<100 Hz), the electrical response is dominated by the formation of the electrical double layer at the electrolyte/electrode interface, followed by an interaction of the film with the analyte (at the kHz region), while at higher frequencies (>100 kHz), the electrical response is dominated by the geometry of the electrodes. Bode diagrams (Figure 4) show all curves differing at low (double electric domain), mid (electrode–solution interface effects), and high (geometric capacitance) frequencies for the various analytes due to the distinct electrical nature of the sensing units. One can note that all impedance spectra fall in the same region at high frequencies (∼1 MHz), as the electrodes have the same geometry. Specifically, IDE1 has a high sensitivity to NaCl solution, taking ultrapure water as a reference, but the response to KCl occurs at low frequencies. Higher dissimilarity to NaCl occurs only at the higher frequency region for IDE4 > IDE3 > IDE2. KCl presents noticeable variation at a lower frequency in contrast to other spectral regions, as it follows IDE4 > IDE3 > IDE2.

**Figure 4:**
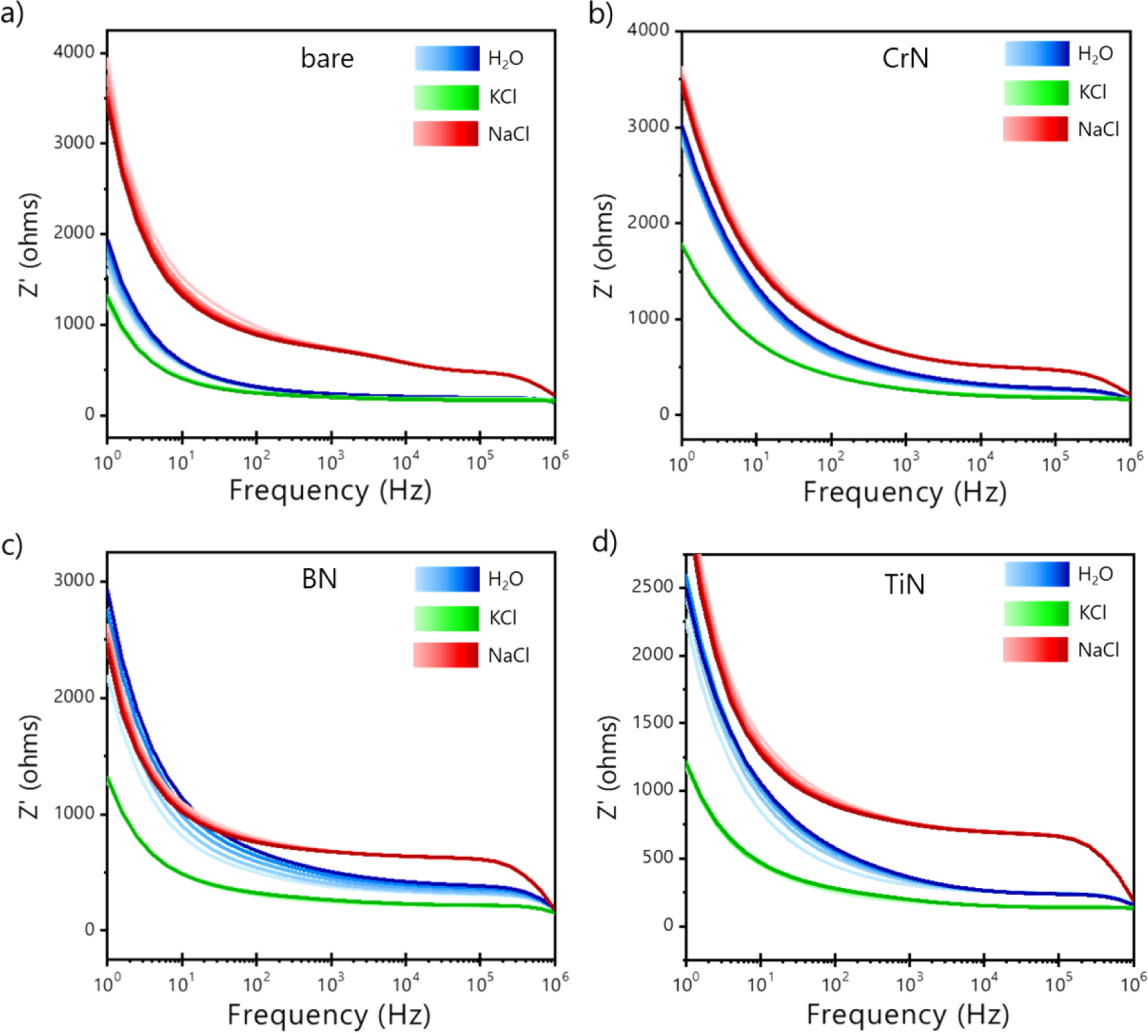
Real impedance vs frequency spectra (Bode diagram) with five electrical measurements for ultrapure water (18.2 MΩ·cm), KCl, and NaCl solutions for: (a) IDE1-nonmodified electrode and electrodes modified with (b) IDE2-CrN, (c) IDE3-BN, and (d) IDE4-TiN films. Measure at flow rate of 15 mL h^–1^.

Figure 5 shows the PCA score plot from raw impedance measurements across the entire frequency range (Figure 4). The ellipses drawn in the plot are the Mahalanobis distance (MD) analysis, with a 95% confidence interval defining the cluster boundaries in the PCA space. MD is a statistical measure quantifying the distance between a point and an elliptical data distribution. Unlike the Euclidean distance, which only considers the direct coordinate differences, the MD considers the correlation between principal components and the variability of the data.^38^ Figure 5 illustrates PC1 explaining most of the variance and effectively distinguishes the formed clusters, demonstrating the e-tongue capability to identify samples with similar structural and chemical properties, positioning NaCl at positive and KCl at negative scores, with the ultrapure water at the origin, while PC2 distinguishes saline solutions from water.

**Figure 5:**
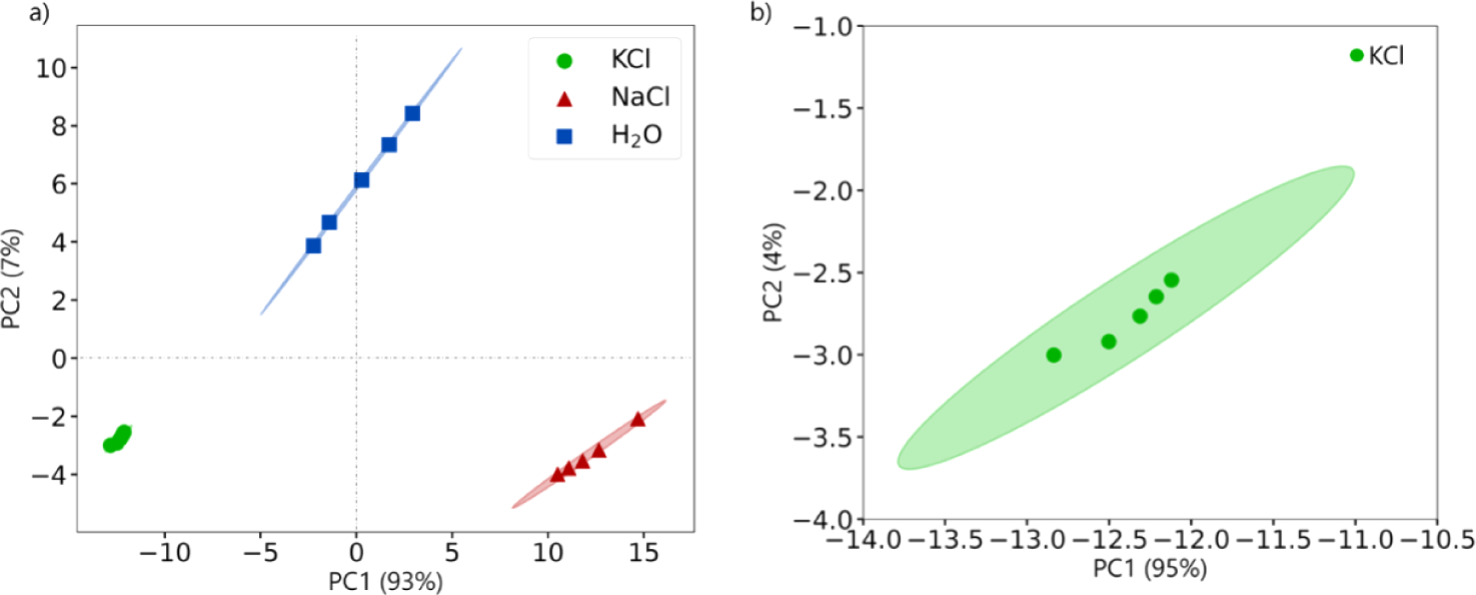
(a) PCA for all independent measurements, derived from raw impedance data across the entire frequency range; (b) increased magnification of the region associated with the KCl.

We chose these analytes due to the size difference between Na^+^ and K^+^ to check whether the system is sensitive enough to detect subtle changes in solutions at the same molar concentration, considering distinct characteristics in the water dynamics caused by the presence of different ions.^39^ The ionic mobility in aqueous systems depends on the charge and size of the ion, modulated by the structure and dynamics of the hydration layers and the solvent involved.^40,41^ The solvation layer around small ions is significantly altered by charge neutralization, and these changes, although less drastic for larger ions (Cl^–^ ∼181 pm > K^+^ ∼138 pm > Na^+^ ∼102 pm) with weaker fields, are also qualitatively different, as larger solutes tend to be more encapsulated by the solvent molecules, with a finite lifetime determined by water–water bonding and hydrogen bond interactions.^41^ Studies show that the free energy and entropy of solvation are continuous functions of their charge and size, with a peak in the solvation entropy of cations as a function of size. Therefore, the mobility of ions in aqueous solutions due to ion size is related to the solvent structure around the species (K^+^ = 7.12 × 10^–8^ m^2^ V^–1^ s^–1^ > Na^+^ = 4.98 × 10^–8^ m^2^ V^–1^ s^–1^),^41^ characterized by the solvation entropy, free energy, and solvation dynamics, which,^40^ although subtle, can be detected here by impedance measurements. Shortly, as K^+^ is larger, its positive charge (+1) is distributed over a larger area, implying a lower charge density, resulting in fewer associated water molecules on the hydration layer and an increased mobility in the aqueous solution. As Na^+^ is smaller, its positive charge (+1) is concentrated in a reduced area, resulting in a firmer, organized hydration layer, which drags more water molecules, thus reducing the mobility of the ions in the aqueous solution. Figures 4 and 5 indicate, each in their own way, that our sensing units can perceive the presence of each ion in the electric field lines of the IDEs. In other words, this occurs because the film deposition affects the surface potential at the electrode–electrolyte interface, which is better noticed at middle frequencies. From Figure 4, one can observe higher impedance values from NaCl, clearly indicating lower conductance when compared with KCl. Therefore, when combined, the impedimetric responses can capture slight variations in the IDE region, thereby helping to build a pattern that identifies the analytes.

Figure 6 shows the parallel coordinate plot from the IDMAP multidimensional projection technique (depicted in the Supporting Information). Although similarity may occur at low frequencies (Figure 6a) for the bare IDE response to ultrapure water and KCl solution or at high frequencies for IDE3 (covered with the BN film) with ultrapure water and NaCl solution (Figure 6c), there is a clear distinction of all samples in most of the frequency range analyzed. That is evidenced by the silhouette coefficient (SC), which measures the quality of data clustering, indicating how well an object fits within a cluster. Overall, SC values close to 1 indicate that the point is well discriminated in its cluster, with values close to 0 suggesting that the point is close to the border between two clusters, and negative values indicate that the point may have been incorrectly distinguished, as it is closer to points in another cluster. Based on the SC, it is observed that the sensing units formed by CrN (SC = 0.94) and TiN (SC = 0.93) films showed better resolution for the tested samples. When the curves in Figure 6 intersect a particular region, that indicates poor separation between samples. Another significant advantage of this method is the potential for performance optimization of each sensing unit in the frequency ranges analyzed, as seen in Figure S3. Except for the higher frequencies of the IDE3 (BN film-coated electrode), the chosen sensing units are effective across the analyzed frequency spectrum. IDEs coated with CrN and TiN films perform better than the bare IDE and the IDE coated with a BN film, covering a broader range of operating frequencies (Table S2). This suggests that each film covering identical IDEs produces distinct electric field lines, enabling the detection of subtle differences in patterns from similar samples. The preliminary analysis with similar ions such as Na^+^ and K^+^ provides a quantitative basis for assessing the system’s accuracy, supporting the reliability of the device in detecting ions with very similar characteristics.

**Figure 6:**
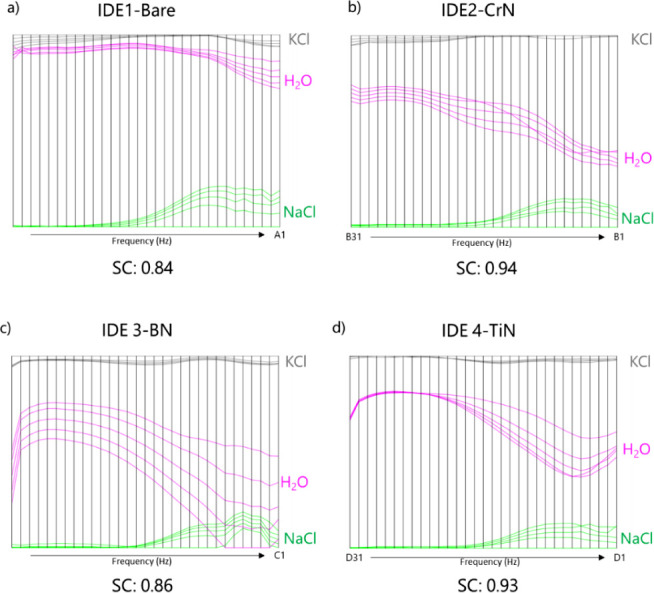
Parallel coordinate plots of IDMAP data projection for the e-tongue, showing the sensor performance across the analyzed frequency bands (as shown in Table S2) for ultrapure water, KCl, and NaCl (1 mmol L^–1^) solutions with four sensing units: (a) IDE1-Bare, (b) IDE2-CrN, (c) IDE3-BN, and (d) IDE4-TiN.

After these results, we challenge our device with 26 distinct soil samples, with a detailed physicochemical analysis in Table S1. Initially, soil samples are analyzed by using the unsupervised IDMAP projection technique, which utilizes the entire magnitude impedance spectrum, where the Fastmap technique is applied to achieve dimensionality reduction. Each spectrum is converted to single-colored points in Euclidean space, as presented in Figure 7, with a silhouette coefficient of 0.801. It is also worth mentioning that clusters that overlap due to a 2D projection remain separate, as zooming in on that region indicates a good separation of all groups formed. Although simple, this is a significant result because it shows the device’s ability to distinguish 26 complex aqueous systems.

**Figure 7:**
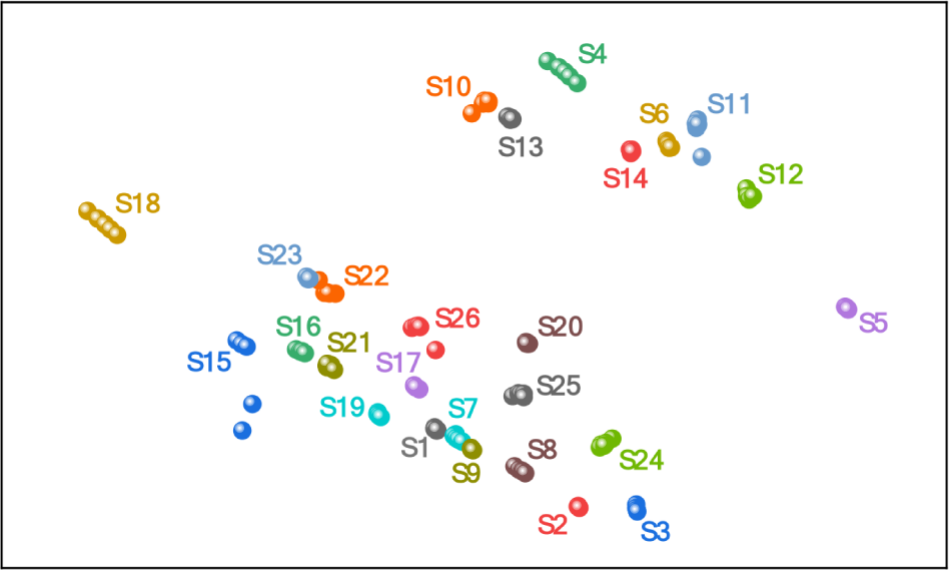
IDMAP plot of magnitude impedance data from 26 soil samples diluted in water.

No pattern recognition could be established despite the discrimination of soil samples through clustering analysis. For this reason, supervised multitask regression analysis is applied to perform the simultaneous determination of four macronutrients (K^+^, Mg^2+^, Ca^2+^, and PO_4_^3–^) using raw impedance data from our microfluidic e-tongue. A total of 23 soil samples, measured in quintuplicate, resulted in 115 impedance spectra that are used to develop the ML model. While 92 spectra are used for training and cross-validation, 23 are reserved as the test data set. Table 1 details the performance of the four regression models (PLS, DT, RF, and XGBoost) evaluated using root mean squared error (RMSE) and the coefficient of determination (*R* ^2^) for training, cross-validation, and testing for all nutrients. PLS presented the highest errors with lower generalization due to a decrease in *R* ^2^ cross-validation values, making it the least reliable model. DT presented a good performance during training and testing but exhibited high errors and a low *R* ^2^ index during the cross-validation experiments, pointing to less reliable future predictions. XGBoost and RF metrics indicate that both are the best models here due to the high accuracy and strong generalization achieved. With a lower error value provided, from this point on, we will only present the RF results, which were trained using up to 50 trees, with bootstrap sampling and out-of-bag estimation enabled, and further evaluated using 10-fold cross-validation.

**Table 1:**
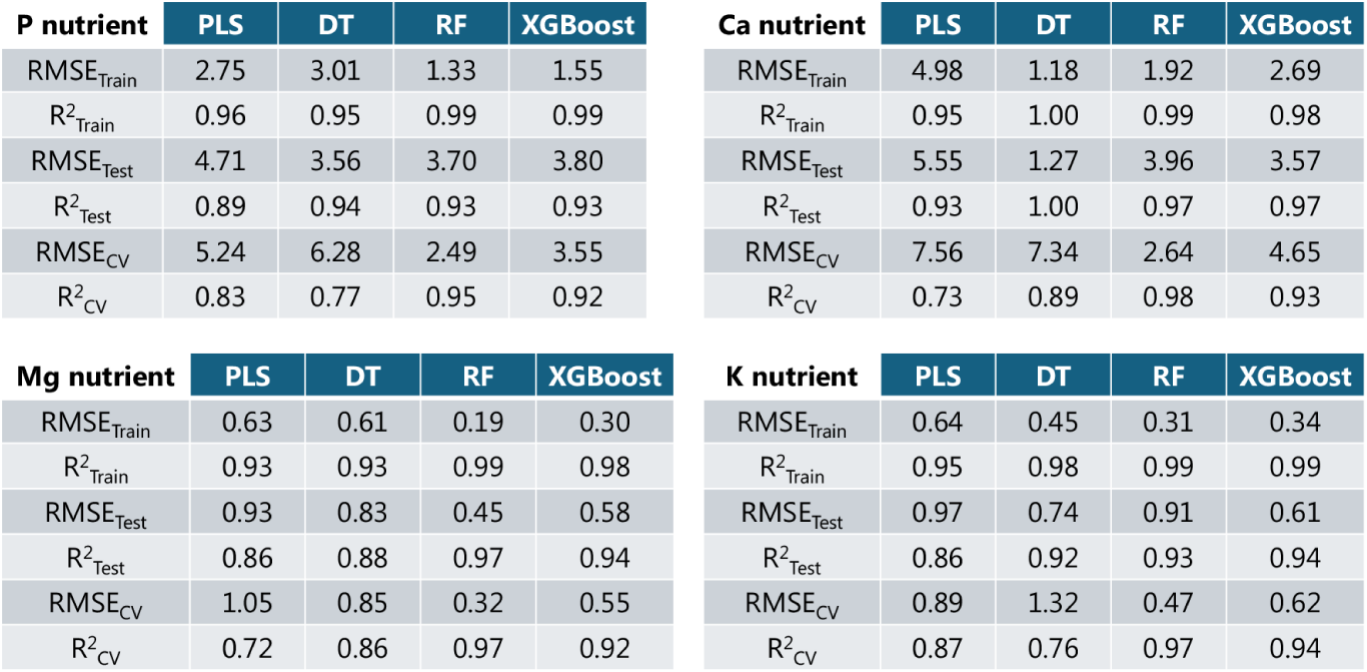
Cross-Validation, Training, and Test Performance Metrics of the PLS, Decision Tree, Random Forest, and XGBoost Regressor Models for the Four Macronutrients

The concentration of four nutrients predicted by the RF multioutput regression model is plotted against the values previously determined using physicochemical methods for both training and test data sets, as shown in Figure 8. Standard deviation values from the training predicted concentrations ranged from 0 to 3.09 (mg dm^–3^), 3.11 (mmol dm^–3^), 0.54 (mmol dm^–3^), and 0.63 (mmol dm^–3^) for PO_4_^3–^, Ca^2+^, Mg^2+^, and K^+^ nutrients, respectively. A high linear profile can be observed (see Table 1), revealing that our device can predict the concentration of the ions present in the soil samples. RMSE values are normalized (NRMSE)^42^ to allow comparisons among the nutrients presenting concentration at different scales. Low NRMSE values are obtained in all cases, indicating a high prediction capability and robust generalization of the RF model. The highest NRMSE values are obtained for P, which is a micronutrient that is difficult to analyze due to its ability to complex with other elements in the soil.

**Figure 8:**
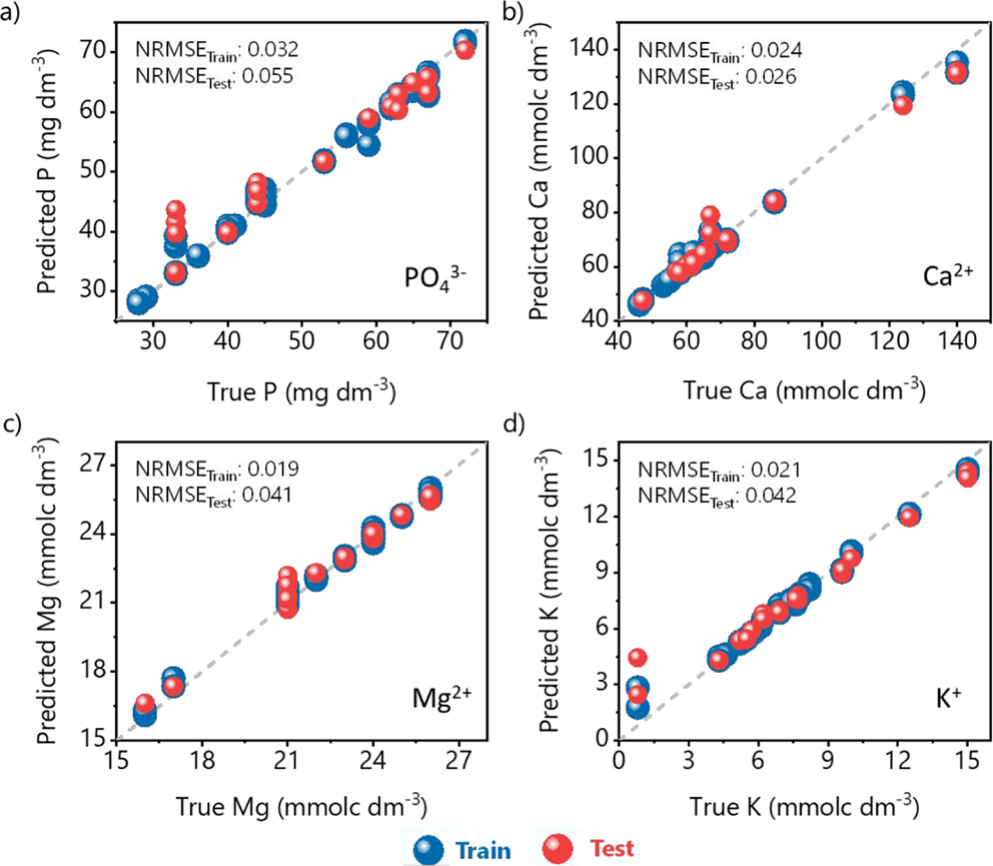
Performance of the RF model. Predicted vs observed values for the multioutput regression model collected in both training and test experiments for the nutrients (a) P, (b) Ca, (c) Mg, and (d) K content. Each point represents a predicted value against its corresponding observed value. The dashed line indicates the agreement between the two values.

In multisensory systems, each sample is measured by an array of cross-sensitive sensors, generating multivariate responses that require proper regression techniques (e.g., PLS, DT, and RF) for analyte prediction. Traditional univariate IUPAC equations are unsuitable for this context because they assume a direct relationship between a single sensor and the analyte. A method proposed by Parastar and Kirsanov^43^ offers a robust multivariate extension of analytical parameters (such as sensitivity, selectivity, and limit of detection), but it is specifically designed for linear models (PLS) that provide interpretable regression coefficients. In contrast, RF is a nonlinear and nonparametric algorithm that does not produce explicit model coefficients. Instead, the mean relative error (MRE) approach, suggested by Kirsanov et al.,^44^ provides a simple, model-independent method for estimating LOD by analyzing the lowest concentration at which the mean relative error (MRE) stabilizes, making it suitable for use with the RF regression model. Thus, yielding 30.7 mg dm^–3^, 46.0 mmol dm^–3^, 16.4 mmol dm^–3^, and 4.81 16.4 mmol dm^–3^ for PO_4_^3–^, Ca^2+^, Mg^2+^, and K^+^ nutrients, respectively. Although our system is capable of measuring concentrations below this point, the concentration values analyzed in this study represent actual variations observed in a cultivated area, ensuring that the LOD reflects practical and agronomically relevant conditions.

As proof of concept, the multioutput RF model is challenged against three blind samples (S7, S13, and S26, limited in number and collected exclusively from the crop control area), deliberately excluded from training/test experiments. It is possible to predict simultaneously the concentrations of the four nutrients using raw impedance data. The accuracy of our model is validated with the true value concentration, obtained with standard physicochemical analysis of soil samples. Figure 9 illustrates both predicted and true concentration values for samples S7, S13, and S26. The overall prediction accuracy is approximately 116% for P, 103% for Ca, 94% for Mg, and 98% for K. Given that real soil samples are complex matrices, this variation in accuracy is acceptable, especially considering that the absolute concentration differences are relatively small, within a few milligrams of dm^–3^. Moreover, the multitask learning model used in this work adds the constraint that all four tasks utilize the same model, which can affect the accuracy of any target.^45^ The Maximum Absolute Error (MAE) values are calculated for each target variable, providing insight into the most significant deviations between predicted and actual values across the samples. Figure S4 shows the violin plot, where the widening of the plot corresponds to a higher density of points at those concentrations; the highest deviation occurred for the P prediction due to the complexity of detecting this species, and the highest data spread is notable for Mg, but once the scales are different for each species, it is challenging to establish a straight correlation. To better comprehend these results, we apply the Lin coefficient (LC), particularly useful in multioutput regression (Figure 9), to evaluate both precision and accuracy, considering how closely predicted values align with observed values along the identity line (*y* = *x*) that varies from −1 to 1. The calculated LC values are 0.77, 0.85, 0.31, and 0.98 for P, Ca, Mg, and K, respectively. It indicates that our microfluidic e-tongue is more sensitive to K, followed by Ca, P, and Mg. This result was expected, as the higher mobility of ions in aqueous solution of K^+^ facilitates its approach to the detection regions, especially in the CrN and TiN films, which exhibit the most significant columnar structures. The low Mg value is influenced by the inherent variability and representativeness of the collected data, obtained from a real crop field area and used to develop the RF model, which does not undermine the purpose of the study and serves as a proof of concept, demonstrating the potential of the approach for nutrient prediction based on spectral data.

**Figure 9:**
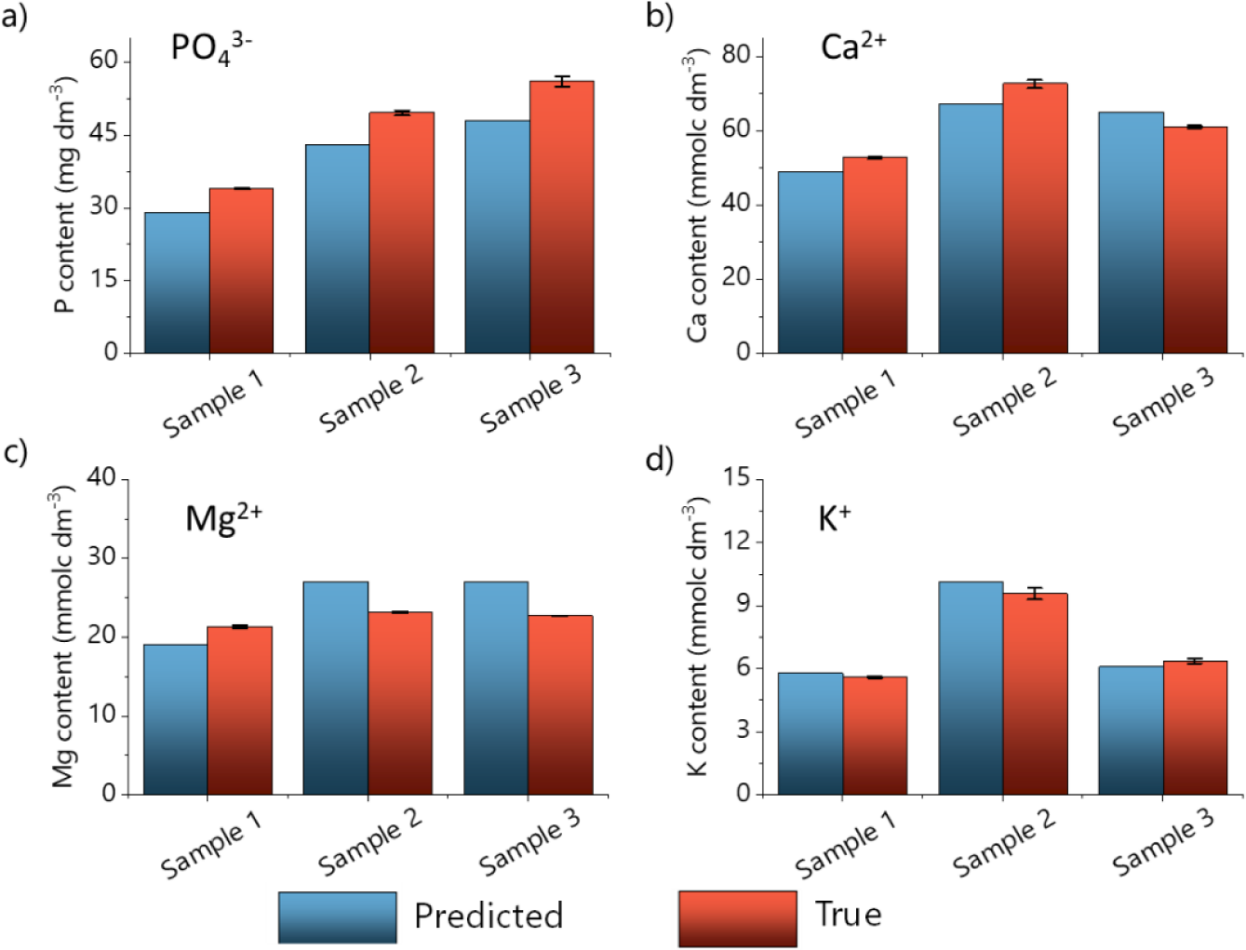
Application of the RF model in blind soil samples. Bar plot shows the predicted and true value concentrations for (a) P, (b) Ca, (c) Mg, and (d) K content for samples S7, S13, and S26.

The K^+^, Ca^2+^, PO_4_^3–^, and Mg^2+^ exhibit distinct characteristics in terms of size and mobility of ions in aqueous solution. This mobility follows the order: K^+^ > Ca^2+^ ≈ Mg^2+^ > PO_4_^3–^. Potassium has higher ion mobility due to its larger size and lower charge; calcium and magnesium have similar mobilities, while PO_4_^3–^ is the smallest and has the higher charge density, resulting in greater hydration and the lowest mobility. These physicochemical differences influence the interaction of the ions with the sensor. In our system, smaller ions exhibit greater proximity to the sensor interface, while larger ions predominantly interact with the surface of the sensing units. Therefore, controlling the proximity and mobility of ions at the electrode–electrolyte interface leads to distinguishable electrical responses that contribute to the ion-specific signal patterns observed. This behavior is consistent with the selectivity observed in our measurements and supports ion differentiation, even in complex matrices such as real soil samples, as corroborated by the computational analysis presented here.

The analysis of the extracted frequencies providing the most relevant information for accurate predictions helps us to identify the optimal performance for this application. By that we ensure a sensor setup tailored for the best performance in real-world applications. Figure 10 shows the 20 most important features obtained from the training data set, where A, B, C, and D represent the sensing units bare, CrN, BN, and TiN, respectively, and the frequencies are ordered from 1 to 31, representing the frequency ranges measured from 1 to 10^6^ Hz (seeTable S2). It is worth noting that the bare electrode (A) contributes little to the model, in contrast to the electrodes modified with CrN and TiN films, with yield importance (sum of importance) of 0.16 and 0.19, respectively. We infer that the higher columnar structures in CrN and TiN films facilitate the approach of some ions to the detection region, generating the observed higher yield importance. The higher frequency region primarily influences the response model, as observed in standard NaCl and KCl solutions. A complementary analysis is performed with the IDMAP projection technique, further plotted with parallel coordinates (Figure S5), with the silhouette coefficient calculated for each feature and represented as colored boxes, in which blue boxes mean strong discrimination (*S* > 0) and red boxes deleterious discrimination (*S* < 0). Similarly, as the supervised RF model presented, the bare electrode presents more red boxes than other sensing units. Although TiN has more blue boxes, the sensing units modified with CrN present blue boxes more filled, which means SC values close to 1, i.e., the highest values.

**Figure 10:**
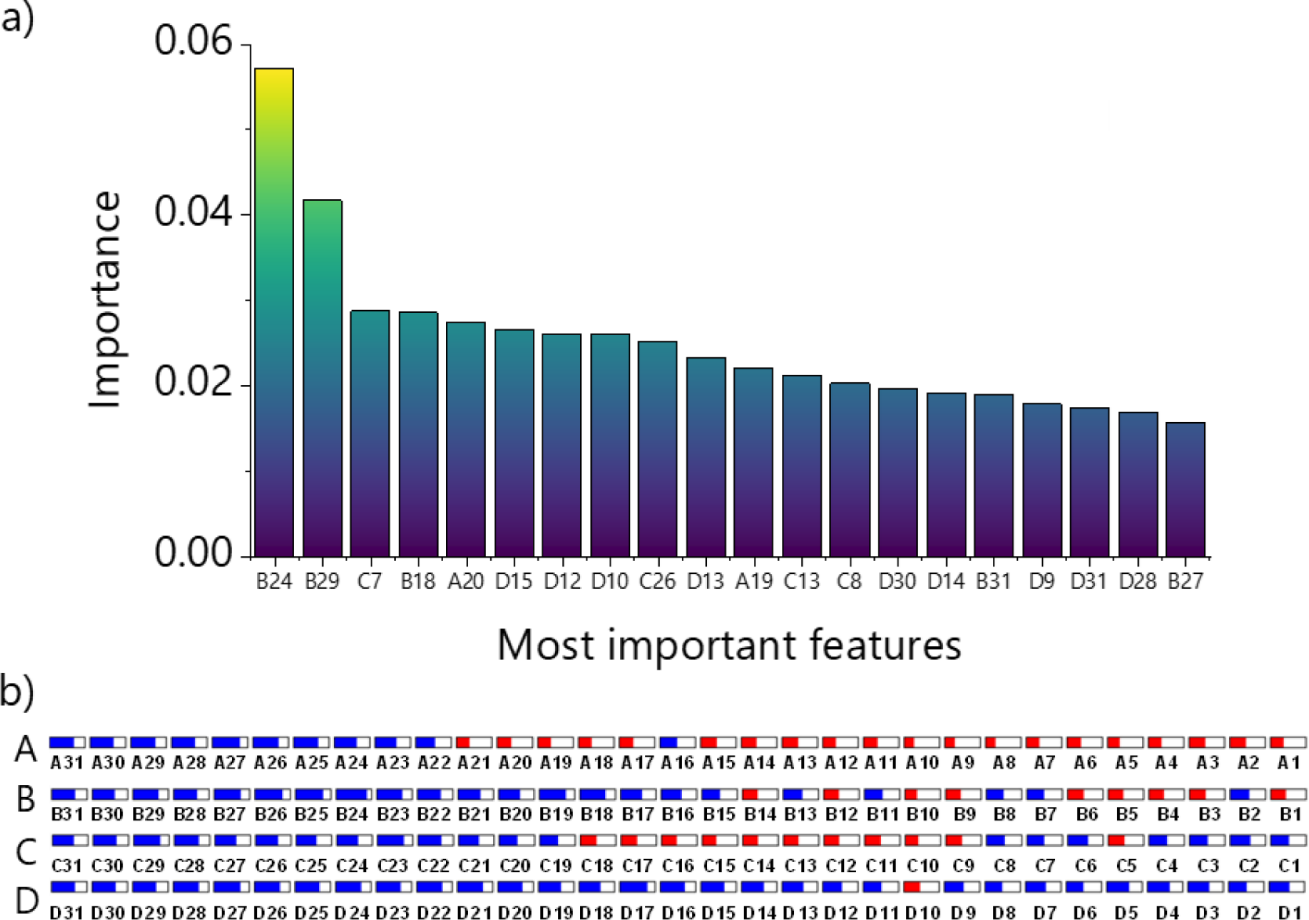
Feature sensory analysis. (a) The 20 most important features determined from the training data set in the RF model. (b) The silhouette coefficient box plot of the parallel coordinate plot was obtained from the IDMAP projection. A: IDE1-bare, B: IDE2-CrN, C: IDE3-BN, and D: IDE4-TiN, and the frequencies are ordered from 1 to 31, representing the frequency ranges measured from 1 to 10^6^ Hz as shown in Table S2. Blue boxes indicate a strong data discrimination since SC > 0, and red boxes are assigned to be deleterious for distinction (SC < 0).

These findings highlight the effectiveness of our approach, mainly using a sensor array that does not rely on the traditional lock-and-key concept. Despite the challenges associated with the sensor setup, low error values are achieved during simultaneous concentration prediction, demonstrating the robustness of our methodology in extracting meaningful information from raw data. This success underscores the potential of frequency-based features for accurate predictions even under less controlled conditions. It is also important to note that these results are from soil samples diluted directly in water (a universal solvent, which facilitates disposal), without pretreatment or the training of specialized personnel.

## Conclusions

Nanometric films obtained through the glancing angle deposition (GLAD) physical vapor technique, deposited onto gold-interdigitated electrodes, successfully form a nitride-based sensor array for soil analysis. Impedance analysis revealed distinct electrical responses across different frequencies in saline solutions, even when chemically similar compounds, such as NaCl and KCl, were compared, highlighting the efficiency and resolution of the selected sensing units. Statistical analysis further confirmed the system’s ability to discriminate between saline solutions based on their ionic composition as well as when applied to real soil samples. These results indicate effective data clustering enabled by the detection materials, which were nanostructured via physical vapor deposition. The sensor setup demonstrated excellent resolution capable of detecting trace concentrations of macronutrients within complex soil matrices. These findings highlight the potential of this e-tongue system in differentiating samples that are diluted in water without pretreatments, supporting green analytical practices and simplifying soil analysis procedures. We emphasize the importance of using statistical and computational methods in a configuration that does not use the lock-and-key concept. This extends the possibility of prolonged device use and provides reliable predictions, improving decision-making in precision agriculture.

## Supplementary Material



## Notes

Instrumentation setup (Figure S1); table of chemical analyses soil samples (Table S1); XPS spectra and deconvolution for of the Oxygen 1s spectra (Figure S2); performance of each sensor across the analyzed frequency bands (Figure S3); violin plot of MAE values (Figure S4); schematic representation of the electrodes and the frequencies used (Table S2); parallel coordinate plot from IDMAP (Figure S5) (PDF)
