# Supplementary material for: Metallic Nitride Microfluidic e‑Tongue: A Novel Selective Approach for the Detection of Macronutrients in Soil
Source: ACS Sens. 2025 Aug 12;10(8):5760–71. doi: 10.1021/acssensors.5c00921 (PMC12379182; doi:10.1021/acssensors.5c00921)
Supplement: Supplementary file 1 [file se5c00921_si_001.pdf]

Supporting Information

Metallic Nitride microfluidic e-tongue: a Novel Selective Approach for the Detection of Macronutrients in Soil

Boeira, C.D.[a*], Leidens, L.M.[a], Costa, E.E.C.[a], Gonçalves, M.H.[a], Perillo, A.S.[a], Ferraz, F.A.[a], Marchi, M.C.[c,d], Shimizu, F.M.[a], Amaral, L.R.[b], Alvarez, F.[a], Riul Jr, A.[a*]

[a]Instituto de Física ‘Gleb Wataghin’ (IFGW), Universidade Estadual de Campinas (UNICAMP), 13083-970, Campinas, SP, Brazil

[b]School of Agronomic Engineering (FEAGRI), Universidade Estadual de Campinas (UNICAMP), 13083-875 Campinas, SP, Brazil

[c]Universidad de Buenos Aires, Facultad de Ciencias Exactas y Naturales, Departamento de Química Inorgánica, Analítica y Química Física, C1428EGA, Buenos Aires, Argentina.

[d]CONICET - Universidad de Buenos Aires, Instituto de Física de Buenos Aires (IFIBA), Centro de Microscopías Avanzadas (CMA), C1428EHA, Buenos Aires, Argentina.

*Email: boeira@unicamp.br and riul@unicamp.br

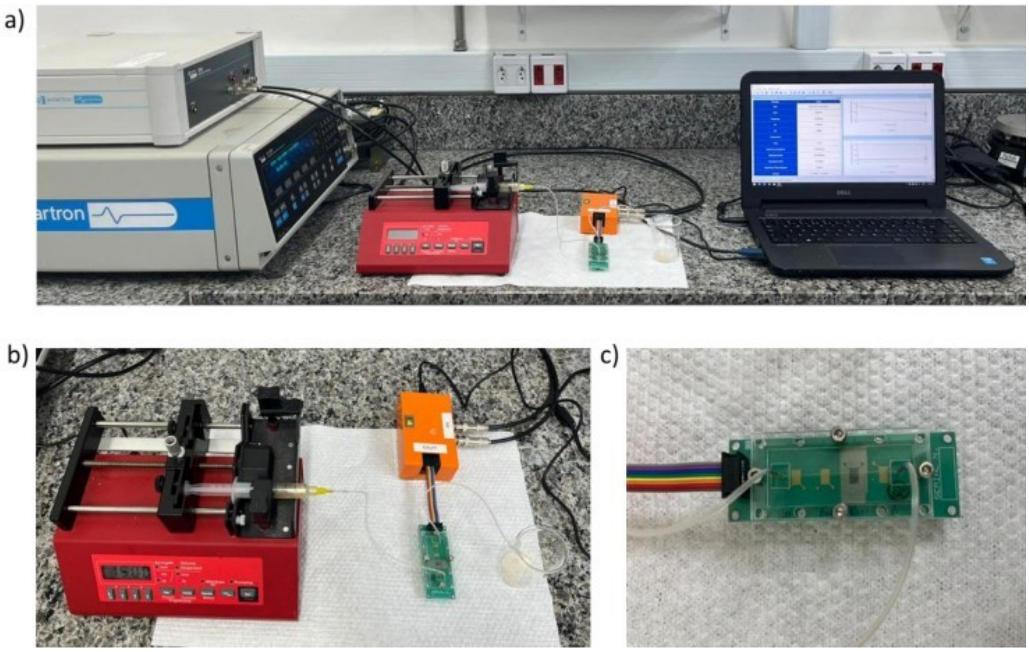

Figure S1: (a-c) Instrumentation setup. (a) on the left instrument Solartron 1260A, followed by syringe pumping, next is a e-tongue device with a multiplexer is connected to an impedance analyser and on the right the computer controlled experiment by a SMaRT 3.3.1 software; (b) magnification of syringe pumping, next is a e-tongue device with a multiplexer; (c) e-tongue: printed circuit board (PCB), encompassing the four collinear detection units with 3 different and microchannel molded in PDMS polymer with mechanical pressure on the plate, keeping only the microchannel that covers the 4 IDEs and inlet and outlet ports mold using a biopsy punch.

Table S1: Table of chemical analyses soil samples

Sample identification	pH	Total Organic Carbon %	P (mg dm^{-3})	Ca (mmolc dm^{-3})	Mg (mmolc dm^{-3})	K (mmolc dm^{-3})	Clay (g dm^{-3})
**S_1**	5.4	21	28	46	22	7.8	409
**S_2**	6.1	25	56	62	23	7.4	421
**S_3**	6.3	23	44	67	21	0.8	412
**S_4**	7.2	24	40	68	24	5.5	428
**S_5**	6.1	28	72	124	16	10	393
**S_6**	5.9	25	29	53	21	4.6	411
**S_7**	6.3	24	29	49	19	5.8	453
**S_8**	6	25	33	62	25	5.5	467
**S_9**	5.8	24	36	54	22	4.6	458
**S_10**	6	26	62	57	22	5.7	473
**S_11**	6	26	40	58	24	4.3	452
**S_12**	5.9	24	41	55	22	6.1	491
**S_13**	6	25	48	65	27	6.1	473
**S_14**	6.1	25	65	66	23	6.9	464
**S_15**	5.7	27	45	58	21	8.2	462
**S_16**	5.8	30	59	65	24	7.6	458

Table S1: Table of chemical analyses soil samples (continued)

Sample identification	pH	Total Organic Carbon %	P (mg dm^{-3})	Ca (mmolc dm^{-3})	Mg (mmolc dm^{-3})	K (mmolc dm^{-3})	Clay (g dm^{-3})
**S_17**	5.4	28	33	62	21	6.2	459
**S_18**	5.2	26	29	55	23	5.8	501
**S_19**	5.9	22	33	47	17	5.4	461
**S_20**	6	25	63	60	24	7.7	457
**S_21**	5.4	31	33	61	26	5.2	527
**S_22**	6	29	67	72	26	9.6	506
**S_23**	5.4	24	44	60	24	6.9	478
**S_24**	6.7	28	67	140	21	15	552
**S_25**	6.1	30	53	86	23	12.5	567
**S_26**	5.5	28	43	67	27	10.1	562

*mmolc: unit of measurement used to express the quantity of matter in soil analysis according to Brazilian soil studies.[45]

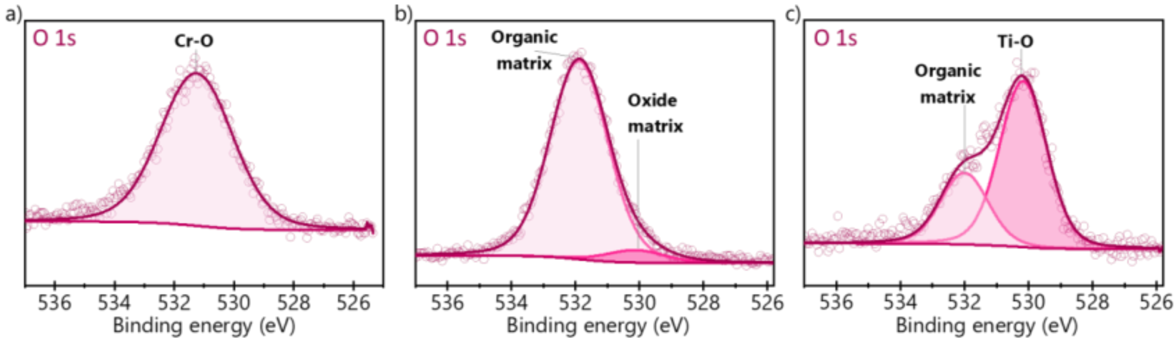

Figure S2: XPS spectra and deconvolution for of the Oxygen 1s spectra to (a) CrN film; (b) BN film; (c) TiN film.

Figure S2 (a-c) show the XPS spectra of emitted photoelectrons for the O 1s binding energy in each film analyzed. The oxygen peak deconvolution analysis for CrN films is shown in Figure S2 (a) where the binding energy of 531.2 eV corresponds to the Cr-O bond. The XPS spectra for oxygen present in the BN film are shown in Figure S2 (b). The O 1s peak is deconvolved into two peaks corresponding to oxide matrix at 530.0 eV and the presence of organic matrices at 531.9 eV, which corresponds to surface impurities. Lastly, the oxygen peak deconvolution analysis is shown in Figure S2 (c). The O 1s peak is deconvolved into two peaks corresponding to Ti-O at 530.0 eV and the presence of organic matrices at 531.9 eV [46].

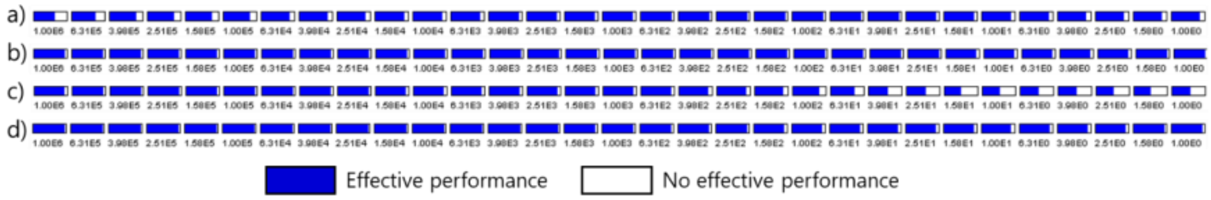

Figure S3: Performance of each sensor across the analyzed frequency bands, (a) IDE1-bare, (b) IDE2–CrN, (c) IDE3-BN and (d) IDE4–TiN.

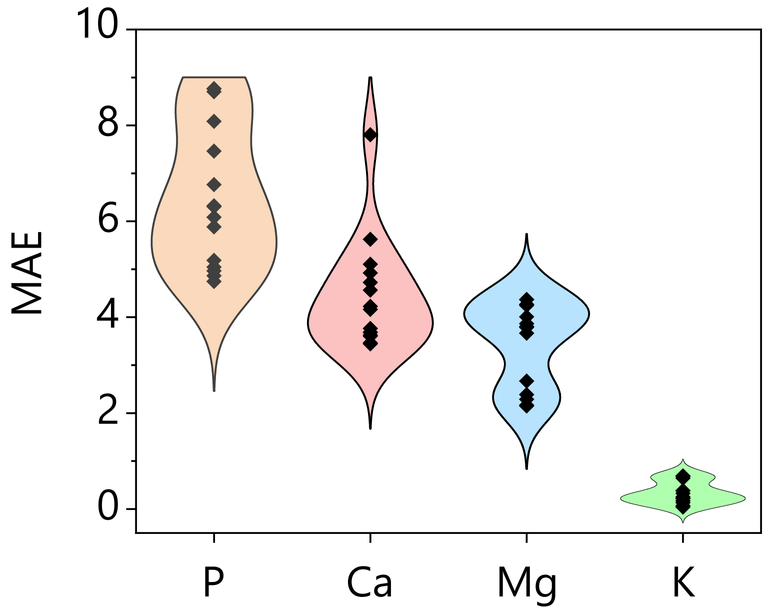

Figure S4: Violin plot of MAE values calculated from prediction of blind soil samples using the RF method against the true concentration values of nutrients from soil determined with conventional physicochemical methods.

Table S2: Schematic representation of the electrodes and the frequencies used, where X from A to D represents the sensors (bare, CrN, BN, and TiN, respectively), and the frequencies are ordered from 1 to 30 within the range of 1 to 10^6 Hz.

XN°	FREQUENCY (HZ)	XN°	FREQUENCY (HZ)	XN°	FREQUENCY (HZ)
**X1**	1.00	**X11**	100	**X21**	10000
**X2**	1.58	**X12**	158	**X22**	15848
**X3**	2.51	**X13**	251	**X23**	25118
**X4**	3.98	**X14**	398	**X24**	39810
**X5**	6.31	**X15**	630	**X25**	63095
**X6**	10	**X16**	1000	**X26**	100000
**X7**	15	**X17**	1584	**X27**	158489
**X8**	25	**X18**	2511	**X28**	251188
**X9**	39	**X19**	3981	**X29**	398107
**X10**	63	**X20**	6309	**X30**	630957
				**X31**	1000000

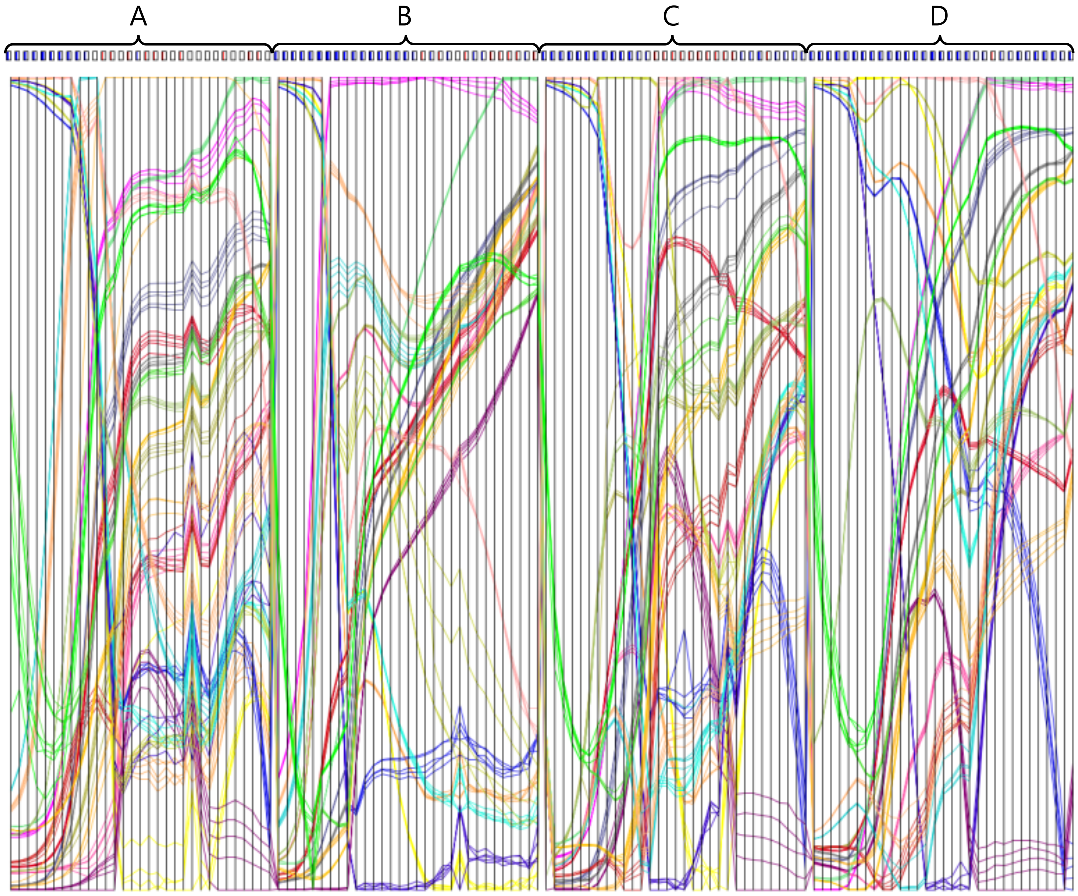

Figure S5: Parallel coordinate plot from IDMAP projection of training dataset of A: IDE1-bare, B: IDE2-CrN, C: IDE3-BN, and D: IDE4-TiN, and the frequencies are ordered from 1 to 31 representing the frequency ranges measured from 1 to 10^6 Hz as shown in the Table S2.
